# Bilateral pleural effusion and interstitial lung disease as unusual manifestations of kikuchi-fujimoto disease: case report and literature review

**DOI:** 10.1186/1471-2466-10-54

**Published:** 2010-11-05

**Authors:** Alberto Garcia-Zamalloa, Jorge Taboada-Gomez, Pilar Bernardo-Galán, Fernandez-Martinez Magdalena, Laura Zaldumbide-Dueñas, Mario Ugarte-Maiztegui

**Affiliations:** 1 Internal Medicine Service. Western Gipuzkoa Clinical Research Unit. Mendaro Hospital. 20850 Gipuzkoa - Spain; 2 Western Gipuzkoa Clinical Research Unit. Mendaro Hospital, 20850 Gipuzkoa - Spain; 3 Internal Medicine Service. Mendaro Hospital., 20850 Gipuzkoa - Spain; 4 Pathology Department, Mendaro Hospital. 20850 Gipuzkoa - Spain; 5 Radiology Service, Mendaro Hospital. 20850 Gipuzkoa - Spain

## Abstract

**Background:**

Kikuchi-Fujimoto's disease (KFD), also called histiocytic necrotizing lymphadenitis, is a rare, idiopathic and self-limited condition usually characterized by cervical lymphadenopathy and fever, most often affecting young patients. Aetiology is unknown. Differential diagnosis includes mainly malignant lymphoma, tuberculous lymphadenitis and systemic lupus erythematosus (SLE), so early diagnosis is crucial. Pleuropulmonary involvement due to isolated KFD has been seldom reported.

**Case Presentation:**

a 32-year-old man, on treatment for iatrogenic hypothyroidism, was admitted due to high grade fever and painful cervical lymphadenopathies. KFD was diagnosed by lymph node biopsy. Some days after admission the patient got worse, he developed generalized lymphadenopathy, bilateral pleural effusion and interstitial lung disease. All of them resolved with prednisone and after two years of following up he remains asymptomatic and without evidence of any other associated disease.

**Conclusion:**

Pleural effusion and interstitial lung disease are very uncommon manifestations of KFD. In our experience, treatment with oral prednisone was effective.

## Background

Kikuchi-Fujimoto's disease (KFD), or histiocytic necrotizing lymphadenitis, was first described in Japan in 1972 as a self-limiting disease mostly affecting the cervical lymph nodes of young individuals, mainly females [1]. In 1982 the first cases of KFD were reported in North America and Europe [2] and the disease is now reported worldwide. Although the aetiology is unknown, it has been suggested to be an apoptotic process mediated mainly by CD8-positive T lymphocytes, and viral or autoimmune factors are believed to be involved [1,3]. Occasionally it may be associated with autoimmune disease, mostly systemic lupus erythematosus (SLE) [3,4]. Enlargement of cervical lymph nodes, fever and leukopenia are the most prominent symptoms, although several other clinical manifestations have been reported [1,3]. The authors report the first case of isolated KFD with interstitial lung disease and bilateral pleural effusion, which also improved quickly with oral prednisone.

## Case Report

**Figure 1:**
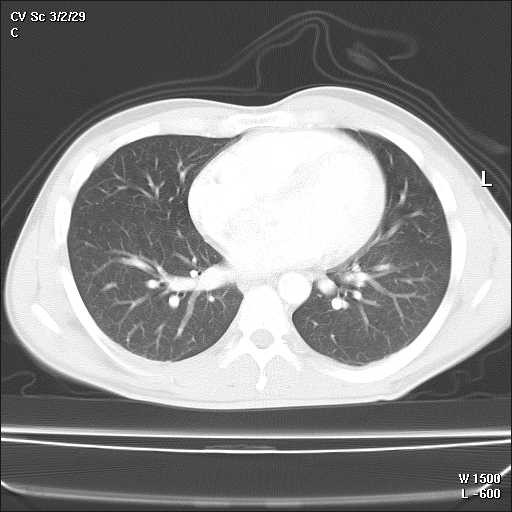
**On admission both lung fields were clear and one isolated mediastinal lymph node was observed**.

**Figure 2:**
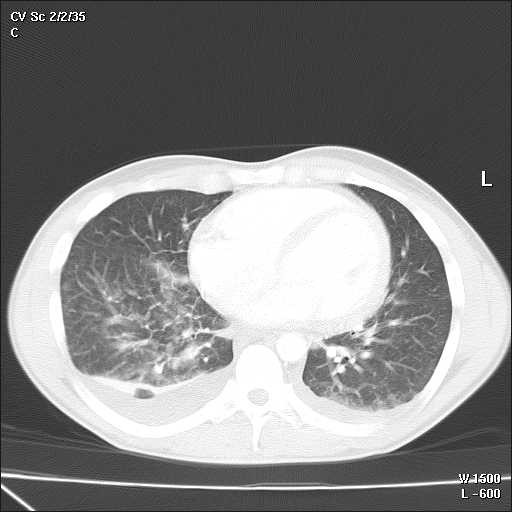
**Some days after admission the patient got worse; and bilateral pleural effusion, interstitial infiltrate in both lungs, and mediastinal and hilar lymphadenopathy developed**.

**Figure 3:**
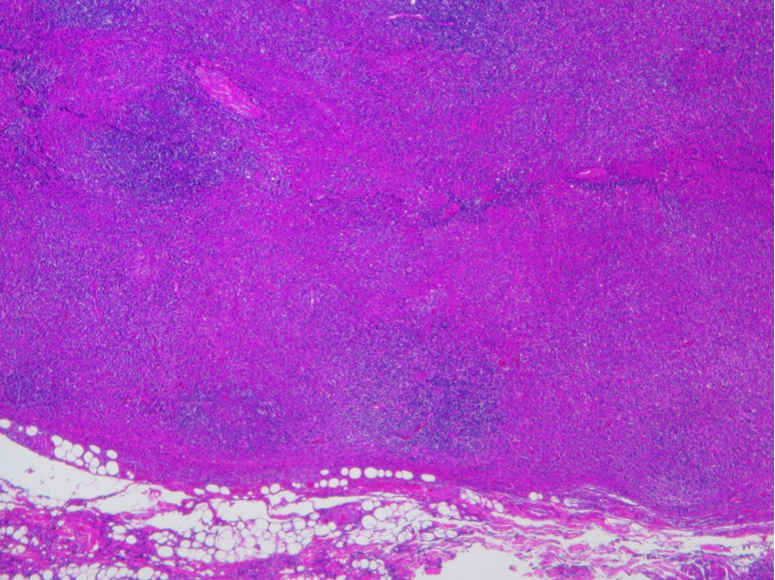
**Peripheral part of the lymph node with cortical and paracortical necrosis, associated with distortion of normal nodal architecture**. Hematoxylin and eosin staining; magnification ×10.

**Figure 4:**
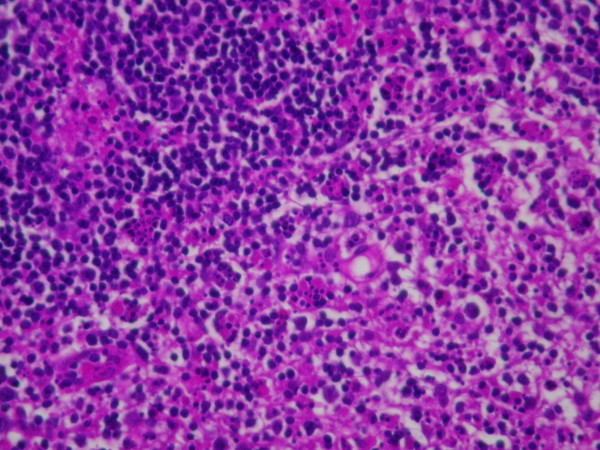
**Non-neutrophilic karyorrhexis**. Histiocytes and plasmacytoid monocytes performing phagocytosis of cellular debris. Hematoxylin and eosin staining; magnification ×60.

A 32-year-old Caucasian man, with a history of hyperthyroidism six months previous due to toxic multinodular goiter, treated with radioactive iodine, who developed iatrogenic hypothyroidism and on initiation of substitutive treatment, was admitted due to persistent fever (39-40°C), malaise and painful cervical lymphadenopathies that had been present for two weeks. Laboratory findings were as follows: haematocrit 34%, haemoglobin 12 g/dl, leukocyte count 3400/mm3 (neutrophils 88.2%, lymphocytes 8.7%, monocytes 2.8%), platelet count 246000/mm3, erythrocyte sedimentation rate 63 mm/h (normal range 0-20 mm/h), alanine transferase (ALT) 176 IU/L (0-40 IU/L), aspartate aminotransferase (AST) 89 IU/L (0-40 IU/L), alkaline phosphatase (AlkP) 176 IU/L (40-129 IU/L) and gamma-glutamyl transpeptidase (GGT) 657 IU/L (10-50 IU/L); serum lactic dehydrogenase (LDH) 1896 IU/L (240-480 IU/L), thyroid-stimulating hormone (TSH) 73 μIU/ml (0.27-4.2 μIU/ml) and thyroxine (T4) 0,25 ng/dl (NR:0.93-1.71). Blood cultures and serologic tests were found to be negative for human immunodeficiency virus (HIV), hepatitis B virus (HBV), hepatitis C virus (HCV), Epstein-Barr virus (EBV), cytomegalovirus (CMV), herpes simplex virus (HSV), *Rubella*, *Toxoplasma*, parvovirus B19, *Yersinia enterocolitica*, *Salmonella and Brucella*. Serum antinuclear antibody (ANA) and rheumatoid factor were also negative. On admission, chest X-ray was clear in both lung fields and computed tomography of the neck, thorax and abdomen revealed only lymphadenopathy affecting bilateral cervical and one mediastinal lymph nodes (Figure 1). Cervical lymph node surgical biopsy was performed on the second day after admission. On macroscopic examination it appeared homogeneous, of medium consistency, dark grey colour and 2 × 1.2 × 1.1 cm in size. The patient's condition worsened over the following days, with high-grade daily fever, increasing size of cervical lymph nodes, the development of axillary lymphadenopathy and mild dyspnoea on exertion, with decreased breath sounds in both lung bases on physical examination. A second computed tomography of the thorax revealed generalized axillary, mediastinal and hilar lymphadenopathy, interstitial infiltrate in both lungs and bilateral pleural effusion (Figure 2). Thoracocentesis was performed with the following results in pleural fluid: pH 7.39 (6.8-7.6), glucose 102 mg/dl (60-100), proteins 3.2 g/dl (0-3), LDH 1694 IU/L (0-200), 50 leukocytes/ml (0-300); stains and cultures for bacteria and mycobacteria were negative and malignant cells were not found on cytologic examination. Cervical lymph node biopsy revealed necrotizing lymphadenitis with prominent areas of cortical and paracortical necrosis and distortion of the nodal architecture (Figure 3), abundant non-neutrophilic karyorrhexis and large numbers of various types of histiocytes at the margins of the necrotic areas, performing phagocytosis of cellular debris. Stimulated lymphocytes and immunoblasts were observed around these areas, along with reduced numbers of plasma cells and no neutrophils (Figure 4). Lymphoma cells were absent and stains and tissue cultures for bacteria, fungi and mycobacteria were negative. Prednisone therapy was started on the 7^th ^day (after receiving negative viral results) at a dose of 1 mg/kg/day, with rapid improvement: the patient became afebrile on day 10, cervical and axillary swelling and tenderness began to decrease, dyspnoea disappeared and respiratory auscultation normalized. The daily dose of thyroxine was slowly increased by the endocrinologist. Prior to discharge, chest X-ray was normal. Tapering doses of prednisone were prescribed throughout the subsequent two months; all biochemical and haematological parameters normalized except for TSH, which reached normal values four months later. After two years of follow-up appointments the patient remains asymptomatic, physical examination and chest X-ray are absolutely normal and serum antinuclear antibodies remain negative.

## Discussion

KFD is a benign disease that usually resolves spontaneously within one to four months, affects all ethnic groups and is more common in young women (4:1 female:male ratio), manifests as localized lymphadenopathy, usually in the cervical region, and is commonly associated with fever and leukopenia [1,5]. The aetiology of KFD remains obscure: a viral pathogenesis is thought to be the most likely candidate due to its self-limiting clinical course and the lack of neutrophilic response [4,6]. It has been reported in association with Epstein-Barr virus, human herpesvirus, human herpesvirus 8, HIV, HTLV1, dengue virus, parvovirus B19, *Yersinia enterocolítica, Bartonella, Brucella, Entamoeba histolytica *and *Toxoplasma *[7,8]. On the other hand, an autoimmune origin has also been suggested due to electron microscopic studies that have identified tubular reticular structures in the cytoplasm of stimulated lymphocytes and histiocytes in patients with KFD, which have also been noted within lymphocytes and endothelial cells of patients with systemic lupus erythematosus and other autoimmune disorders, so it is possible that KFD may represent an exuberant T-cell-mediated, self-limited immune response to a variety of non-specific stimuli in genetically susceptible individuals [9].

The differential diagnosis of lymph node enlargement in patients with the clinical signs of KFD includes mainly tuberculous and other infectious types of lymphadenitis, and malignant lymphoma [4]. The diagnosis is confirmed by lymph node biopsy, when histopathology reveals necrotizing lymphadenitis restricted to the cortical and paracortical areas, with partial or complete loss of follicular architecture, marked karyorrhexis and absence of neutrophils, granulomatous reaction or lymphoma cells [5]. Most KFD cases improve within a six-month period [3], and a wide range of recurrence rates have been reported, from 4% to 27% [10-12].

Kikuchi-Fujimoto disease has occasionally been described in association with simultaneous SLE [4,5], Sjögren syndrome [13], antiphospholipid syndrome [4,14], relapsing polychondritis [15] or even autoimmune hepatitis [16]. On the other hand, unusual manifestations of isolated KFD include axillary and mesenteric lymphadenopathy, splenomegaly, parotid gland enlargement, skin rashes, arthralgias, myalgias, aseptic meningitis, bone marrow haemophagocytosis and liver dysfunction [3]. Patients with arthritis [17,18] or bilateral panuveitis [19] have been reported on occasion. KFD has seldom been reported in association with pulmonary involvement [20] and most such cases have been in patients who developed SLE [4]. Wilkinson et al. described a 41-yr-old lady diagnosed with KFD who subsequently developed polymyositis with pulmonary involvement [21]. Meanwhile, Kucukardali et al. reported one case of fatal pulmonary haemorrhage due to KFD upon post-mortem examination and three cases who developed KFD after transplantation and died of respiratory failure [3]. Finally, one case of KFD associated with cryptogenic organizing pneumonia has been reported recently [22]. In the present case, the patient developed isolated KFD with pleuropulmonary involvement due to interstitial lung infiltrate and pleural effusion, both bilateral and with rapid improvement and resolution with oral prednisone. The simultaneous hypothyroidism of the patient should not be ignored, nor its possible clinical co-expression. However, its presence several months before the development of KFD and even after the clinical and analytical resolution of the latter, combined with the absence of any other clinical symptoms related to thyroxine defficiency and the rapid development and improvement of the pleuropulmonary manifestations with prednisone, suggest an inflammatory rather than an endocrine aetiology.

In conclusion, pleuropulmonary involvement in patients with KFD has seldom been reported. We describe a patient with isolated KFD in whom bilateral pleural effusion and an interstitial lung pattern developed. Clinical and radiological responses to prednisone were excellent.

## Disclosures: Conflicts of interests

The authors declare that they have no competing interests.

## Authors' contributions

AGZ has contributed providing the clinical data, he has performed the literature review and he has written the main manuscript. He was involved in revising the manuscript critically for important intellectual content, and gave final approval of the version to be published. JTG was involved in revising the manuscript critically for important intellectual content, making substantial contributions and gave final approval of the version to be published. PBG was involved in revising the manuscript critically, and gave final approval of the version to be published. MFM was involved in revising the manuscript critically, and gave final approval of the version to be published. LZD provided the histopathological figures. She was involved in revising the manuscript critically, and gave final approval of the version to be published. MUM provided the Computed Tomography images and was involved in revising the manuscript critically, and gave final approval of the version to be published.

## Consent

Written informed consent was obtained from the patient for publication of this case report and any accompanying images. A copy of the written consent is available for review by the Editor-in-Chief of this journal.

## Pre-publication history

The pre-publication history for this paper can be accessed here:

http://www.biomedcentral.com/1471-2466/10/54/prepub
